# Prognostic impact of steatosis in the clinical course of chronic HCV infection—Results from the German Hepatitis C-Registry

**DOI:** 10.1371/journal.pone.0264741

**Published:** 2022-06-16

**Authors:** Monika Rau, Peter Buggisch, Stefan Mauss, Klaus H. W. Boeker, Hartwig Klinker, Tobias Müller, Albrecht Stoehr, Jörn M. Schattenberg, Andreas Geier

**Affiliations:** 1 Medizinische Klinik II, Universitätsklinikum Würzburg, Würzburg, Germany; 2 ifi-Institute for Interdisciplinary Medicine, Hamburg, Germany; 3 Center for HIV and Hepatogastroenterology, Düsseldorf, Gernamny; 4 Center of Hepatology, Hannover, Gernamny; 5 Charité Campus Virchow-Klinikum (CVK), Berlin, Gernamny; 6 Metabolic Liver Research Program, University Medical Centre Mainz, Mainz, Germany; Medizinische Fakultat der RWTH Aachen, GERMANY

## Abstract

**Background:**

Liver steatosis is often observed in chronic HCV infection and associated to genotype or comorbidities. NAFLD is an important risk factor for end-stage liver disease. We aimed to analyse the course of NAFLD as a concomitant disease in a cohort of HCV patients.

**Methods:**

The German Hepatitis C-Registry is a national multicenter real-world cohort. In the current analysis, 8789 HCV patients were included and separated based on the presence of steatosis on ultrasound and/or histology. Fibrosis progression was assessed by transient elastography (TE), ultrasound or non-invasive surrogate scores.

**Results:**

At the time of study inclusion 12.3% (n = 962) of HCV patients presented with steatosis (+S) (higher rate in GT-3). Diabetes mellitus was more frequent in GT-1 patients. HCV patients without steatosis (-S) had a slightly higher rate of fibrosis progression (FP) over time (30.3%) in contrast to HCV patients +S (26%). This effect was mainly observed in GT-3 patients (34.4% vs. 20.6%). A larger decrease of ALT, AST and GGT from baseline to FU-1 (4–24 weeks after EOT) was found in HCV patients (without FP) +S compared to -S. HCV patients -S and with FP presented more often metabolic comorbidities with a significantly higher BMI (+0.58kg/m^2^) compared to patients -S without FP. This was particularly pronounced in patients with abnormal ALT.

**Conclusion:**

Clinically diagnosed steatosis in HCV patients does not seem to contribute to significant FP in this unique cohort. The low prevalence of steatosis could reflect a lower awareness of fatty liver in HCV patients, as patients -S and with FP presented more metabolic risk factors.

## Introduction

Liver steatosis is frequently observed in patients with chronic hepatitis C virus (HCV) infection, occurring in approximately 50% [1], and the presence and severity of steatosis is an important indicator for progressive liver disease [2]. Non-alcoholic fatty liver disease (NAFLD) is characterized by an excess hepatic fat accumulation, strongly associated with insulin resistance and the metabolic syndrome, and has an estimated prevalence of 20–30% in the general population [3]. Recently, the new term Metabolic Dysfunction-Associated Fatty Liver Disease (MAFLD) has been proposed to emphasize this association [4]. Due to its rising prevalence NAFLD represents a major health concern and it will replace HCV as the leading indication for liver transplantation in the near future in the US [5].

Up to 86% of HCV GT 3 patients show some degree of hepatic steatosis that is associated with serum viral load and reversible with achievement of sustained virological response rate (SVR) [6].

In the great majority of non-GT 3, particularly GT 1 infected patients hepatic steatosis is linked to features of the metabolic syndrome such as BMI, visceral obesity, insulin resistance, type 2 diabetes mellitus (DM), low HDL-cholesterol levels and is therefore termed “metabolic steatosis” [2]. HCV *per se* induces insulin resistance which in turn leads to hepatic steatosis and aggravates insulin resistance. Thus, insulin resistance can be both a direct consequence of HCV infection and a result of NAFLD and vice versa [7]. Additionally, genetic background of the host has an important impact on the development of NAFLD in chronic HCV infections such as I148M variant of *PNPLA3* and the rs58542926 polymorphism in *TM6SF2* [1,8]. In the pre-DAA antiviral treatment era concomitant metabolic steatosis was associated with lower SVR to interferon-based treatment with the exception of GT 3 infection [1].

Concomitant NAFLD or so called “metabolic steatosis” in HCV patients induces higher degree of fibrosis and an increased risk for hepatocellular carcinoma (HCC) [6]. A large number of prospective or cross-sectional studies analysed the impact of steatosis as an individual risk factor for fibrosis progression in HCV infection [9]. In a meta-analysis including 3068 HCV-infected individuals liver fibrosis was independently associated with steatosis and liver inflammation [10]. However, fewer studies found a genotype-dependency of this association of steatosis and fibrosis [11]. The risk factors of fibrosis progression are similar in HCV-related liver disease and in NASH. For both liver diseases increased BMI, type 2 DM and age are associated to fibrosis progression [2].

Experimental as well as clinical evidence show an association between HCV-related steatosis and the risk for HCC [12–14].

Diagnosis of NAFLD in chronic HCV patients can be difficult. The presence of hepatic steatosis or steatohepatitis is needed to define NAFLD in HCV patients after exclusion of alcoholic fatty liver disease by detailed patient report [15]. Additionally, histological findings with specific inflammatory and fibrosis pattern can differentiate NASH from HCV [2].

In this study we put the focus on the clinical course of NAFLD in HCV patients. The aim of this study was to evaluate the course of NAFLD in a large, prospectively enrolled nation-wide registry cohort (the German Hepatitis-C Registry). Fibrosis progression based on the combination of TE, ultrasound and noninvasive surrogate scores were analysed together with clinical endpoints such as SVR and HCC prevalence in HCV patients with and without hepatic steatosis.

## Methods

### Patient characteristics

The German-Hepatitis C Registry (Deutsches Hepatitis C-Register, DHC-R) is an ongoing, prospective, multicentre, observational cohort study with over 15600 patients enrolled. The DHC-R is registered at the Federal Institute for Drugs and Medical Devices (BfArM; number 2493) and in the German Clinical Trials Register (DRKS; ID DRKS00009717) [16]. The study protocol was implemented in accordance with Good Clinical Practice Guidelines and the Declaration of Helsinki. The study was reviewed and approved by Institutional Review Board (Ethics Committee of Ärztekammer Westfalen-Lippe; reference number 2014-395-f-S). All patients had to give written informed consent before enrolment in the registry. Patient data were collected by a web browser based Electronic Data Capture (EDC) system and data quality was analysed by plausibility checks and on site monitoring [17].

To analyse the clinical course of NAFLD in the DHC-R two patient cohorts were defined as following. Patients with steatosis (+S) were diagnosed either by ultrasound and/or by liver histology (ultrasound (n = 944), liver histology (n = 13) or both (n = 5)) after exclusion of significant alcohol consumption. Patients without steatosis (-S) had no known liver steatosis and no reported steatosis by ultrasound or liver histology. Patients without available ultrasound or liver histology were classified as -S (2031/7827). Exclusion criteria were an abusive alcohol consumption and/or a regular alcohol consumption for men > 40g/d and women > 30g/d as reported in the eCRF. According to the study protocol diagnosis of DM includes patients with type 1 and type 2 DM with 90% type 2 DM (type 1 DM n = 92, type 2 DM n = 772). Only patients with data of baseline visit and one follow-up (FU) visit (up to 24 weeks after EOT) were included in the analysis. Fibrosis progression was defined by an increase at least in one of the following diagnostic procedures ultrasound, transient elastography (TE) or laboratory-based sum scores as available between baseline visit and FU visit. By the majority of patients the FU visit was mostly been done up to one year after EOT. In more detail progression of fibrosis was diagnosed either by newly diagnosed sonographic findings of cirrhosis in the FU visit (n = 231) by TE with raising stiffness (Fibroscan: increase >7.2kPa with normal baseline value or increase by >2kPa with baseline value >7.2kPa (n = 97); ARFI: increase >1.3m/s with normal baseline value or increase >0.3m/s with baseline value >1.3m/s (n = 16)) or by progression in a combined fibrosis staging score (n = 97) that included progression of fibrosis stage either in histology or in categorized Fibroscan measurements in kPa (≤7 = ’F0-F1’; >7 - ≤ 9.5 = ’F2’; >9.5 –<12.5 = ’F3’; ≥12.5 = ’F4’) or in categorized ARFI measurements in (m/s) (<1.27 = ’F0-F1’; ≥1.27 - <1.72 = ’F2’). An increase of a non-invasive fibrosis score based on laboratory findings was defined as following (APRI; progression from <0.7 to ≥0.7 (n = 295), FIB-4; progression from <1.45 to ≥1.45 and ≥1.45 to >3.25 (n = 2315) and NAFLD fibrosis risk score (-1,675 + 0,037 × age + 0,094 × BMI + 1,13 × insulin resistance or diabetes (yes = 1, no = 0) + 0,99 × AST/ALT Ratio– 0,013 × thrombocytes– 0,66 × albumin); progression from < -1.455 to ≥ -1.455 and ≥ -1.455 to > 0.676 (n = 356)). According to the study protocol histology was only available to the time point of study inclusion and therefore fibrosis progression based on histology could not be included in the data analysis. A data extract of 8789 HCV patients was analysed in this study. Detailed patient characteristics are described in the results.

### Statistical analysis

The data were analysed based on a data base extract on behalf of Leberstiftungs‐GmbH Deutschland. This analysis includes data through 15 July 2018. Fisher’s exact test was used to compare hepatic steatosis, fibrosis progression, DM, hypertension, dyslipidemia, SVR and HCC occurrence in different genotypes. A two-sided T-test was used for comparison of two paired groups, e.g. for absolute values of TE analysis. Changes of serum liver enzymes from BL to EOT were analysed by Wilcoxon signed rank test. The pairwise comparison of groups for changes in liver enzymes from BL to FU1 was performed by a two-sided Median test. The difference in BMI between different patient groups (+S and -S +/- fibrosis progression) together with ALT elevation was analysed by Anova.

P values < 0.05 were considered statistically significant. Statistical analyses were performed using SPSS version 26.

## Results

### Cohort characteristics

In total, 8,789 HCV patients were included in this analysis. 962 patients (11.0%) were diagnosed with steatosis (+S) and 7,827 (89%) without steatosis (-S). In this real-world cohort the diagnosis of steatosis was assessed either by ultrasound (n = 944), liver histology (n = 13) or both (n = 5). The actual frequency of hepatic steatosis diagnosis in this population is lower than the expected NAFLD prevalence in the general population as well as the previously described frequency of steatosis in other HCV patient cohorts. In patients with GT 3 infection prevalence of steatosis (n = 199) was significantly higher compared to non-GT 3 (n = 763) patients (16.7% vs. 10.0%; p<0.001). All patients received antiviral treatment from baseline visit to FU visit. A large majority received DAA treatment (-S 98.8%; +S 99.1%) and only a minority had Interferon +/- Ribavirin (-S 1.2%; +S 0.9%). Further patient characteristics at BL are depicted in **Table 1**.

**Table 1:** Baseline characteristics of HCV patients including all genotypes.

Patient characteristics % (n)			
*All Gentoypes*	-S (n = 7827)	+S (n = 962)	p-values
**sex m/f**	60 (4597)/ 40 (3230)	60 (580)/ 40 (382)	0.367
**age (y) (MW±SD)**	53.0 ± 12.8	52.8 ± 11.7	0.716
**BMI kg/m^2^ (MW±SD)**	25.6±4.5	28.3±5.6	< 0.001
**Hypertension % (n)**	22.6 (1769)	27.9 (268)	< 0.001
**DM % (n)**	9.4 (734)	13.4 (129)	< 0.001
**Hyperlipidemia % (n)**	2.1 (163)	4.6 (44)	< 0.001
**ALT (U/L) (MW±SD)**	85.7±75.9	94.9±77.3	<0.01
**AST (U/L) (MW±SD)**	67.2±49.5	69.7±47.9	0.154

HCV patients (all GT) +S had a significantly higher BMI compared to patients -S (BMI mean ± SD: +S 28.3±5.6kg/m^2^ vs. -S 25.6±4.5kg/m^2^; p<0.001). This result was likewise observed when analysing only GT 1 patients +S vs. GT 1 patients -S or GT 3 patients +S vs. GT 3 patients -S (**Table 2**). However, no difference in BMI was observed for GT 1 patients (+S and -S) vs. non-GT 1 patients (BMI GT 1: 26.0±4.8kg/m^2^ vs. non-GT 1 25.9±4.7kg/m^2^; p = 0.13). Hypertension, DM and hyperlipidemia occurred significantly more often in patients +S including all genotypes as well as in patients with GT 1 +S compared to GT1 -S (**Table 2**). GT 1 patients had higher frequency of DM and hypertension compared to non-GT 1 patients (DM: GT 1: 10.5% vs. non-GT 1: 7.6%, p < 0.001; hypertension: GT 1: 26.6% vs. non-GT 1: 17.2%, p<0.001), but no significant difference in baseline BMI and hyperlipidemia. ALT serum levels were significantly higher in patients (all genotypes) +S compared to patients–S (+S 94.9±77.3 U/L vs.–S 85.7±75.9 U/L, p < 0.01). No significant difference between serum ALT and AST was observed for GT1 or GT 3 patients +S compared to GT1/GT3 patients -S (**Table 2**).

**Table 2:** Baseline characteristics of HCV GT 1 and GT 3 patients.

Patient characteristics % (n)			
*Genotype 1 (GT1)*	-S (n = 6082)	+S (n = 661)	p-values
**BMI kg/m^2^ (MW±SD)**	25.6±4.5	28.5±5.6	< 0.001
**Hypertension % (n)**	24.9 (1514)	30.1 (199)	< 0.01
**DM % (n)**	10.0 (608)	15.0 (99)	< 0.001
**Hyperlipidemia % (n)**	2.2 (134)	5.1 (34)	< 0.001
**ALT (U/L) (MW±SD)**	81.7±75.9	86.5±67.5	0.108
**AST (U/L) (MW±SD)**	65.3±47.7	65.9±42.0	0.778
***Genotype 3 (GT3)***	**-S (n = 991)**	**+S (n = 199)**	
**BMI kg/m^2^ (MW±SD)**	25.8±4.7	27.5±5.7	< 0.001
**Hypertension % (n)**	12.3 (122)	14.6 (29)	0.414
**DM % (n)**	6.6 (65)	7.0 (14)	0.757
**Hyperlipidemia % (n)**	1.5 (15)	2.0 (4)	0.542
**ALT (U/L) (MW±SD)**	113.0±93.3	118.6±94.5	0.439
**AST (U/L) (MW±SD)**	83.3±59.2	81.4±57.3	0.704

### HCV patients without steatosis and GT 3 had more fibrosis progression

**Fig 1:**
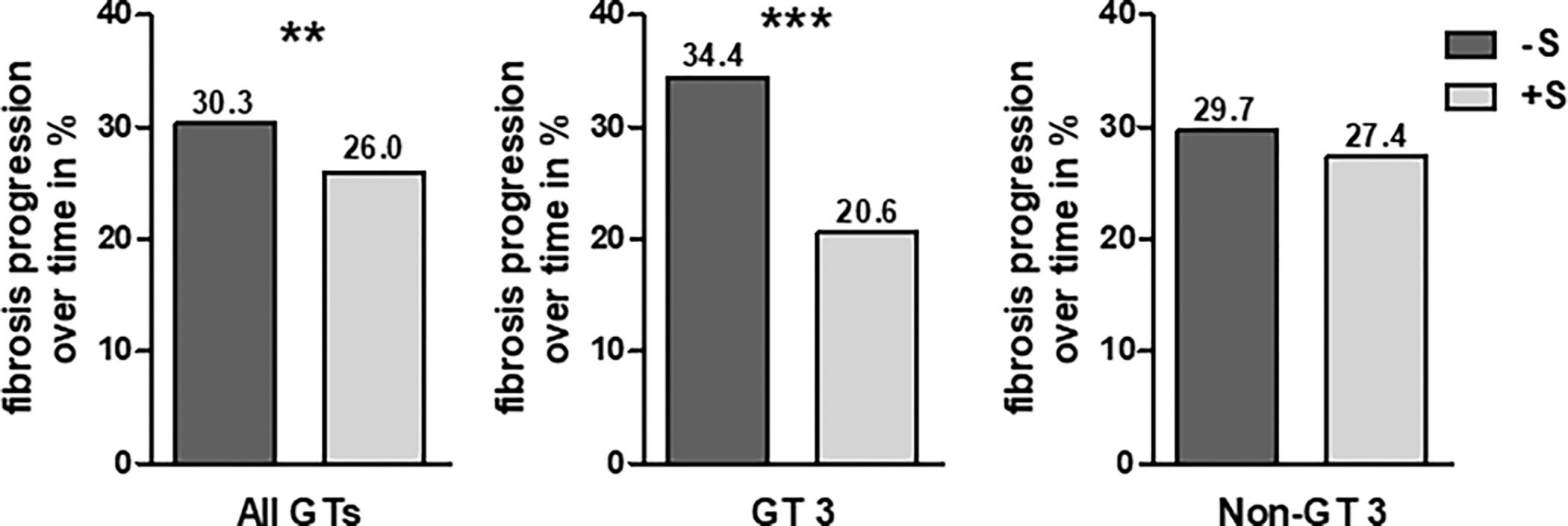
Rate of fibrosis progression over time in % for patients with steatosis (+S) at baseline and without steatosis (-S) at baseline. ** = p < 0.01, *** = p < 0.001.

Fibrosis progression over time was defined by an increase at least in one of the following diagnostic procedures transient elastography (TE) (n = 113, 4.3%), ultrasound (n = 231, 8.8%) or laboratory-based sum scores as available (n = 2275, 86.9%) between baseline visit and one follow-up (FU) visit. By the majority of patients the FU visit was mostly been done up to one year after EOT. Interestingly, HCV patients -S (30.3%, 2,369/7,827) had a slightly higher fibrosis progression rate over time compared to HCV patients +S (26%, 250/962, p<0.001), **Fig 1**. This effect was even more pronounced in GT 3 patients -S (34.4%, 341/991) compared to patients +S (20.6%, 41/199, p<0.001). In non-GT 3 patients no significant difference was observed for patients -S (29.7%, 2028/6836) and patients +S (27.4%, 209/763, p = 0.195). Concomitant liver steatosis showed no increase of fibrosis progression in this real-world cohort.

In a smaller patient cohort (n = 313) TE measurements were available at BL and FU1 (up to 24 weeks after EOT). Elevation in liver stiffness by TE (>7.2kPa) was measured at BL, but no difference was observed in patients +S (59.6%) and -S (56.1%). TE measurements decreased over time and after antiviral treatment. A significant decrease in mean stiffness was observed for HCV patients -S with (+) and without (-) fibrosis progression from BL to first follow-up (FU1) visit (-S—fibrosis progression: 12.6 ± 8.4kPa (BL), 8.4 ± 7.1kPa (FU1); -S + fibrosis progression: 20.0 ± 13.7kPa (BL), 15.3 ± 11.6kPa (FU1); p<0.001 respectively). HCV patients +S—fibrosis progression had also a significant change in liver stiffness from BL to FU1 (+S—fibrosis progression: 9.8 ± 4.1kPa (BL), 7.1 ± 2.7kPa (FU1); p<0.01; n = 19). But no statistically significant change in liver stiffness from BL to FU1 was observed for HCV patients +S + fibrosis progression (+S + fibrosis progression: 19.0 ± 18.5kPa (BL), 9.4 ± 3.9kPa (FU1); p = 0.076; n = 12). The decrease in liver stiffness in patients -S +/- fibrosis progression and +S–fibrosis progression probably shows an effect of antiviral treatment and especially a decrease of necroinflammation. In HCV patients +S + fibrosis progression concomitant NAFLD might hinder the statistically significant difference. Repeated TE measurements showed a decrease in liver stiffness after antiviral treatment with the exception of HCV patients with concomitant liver steatosis.

### Liver enzymes were higher in HCV patients with steatosis over time

**Fig 2:**
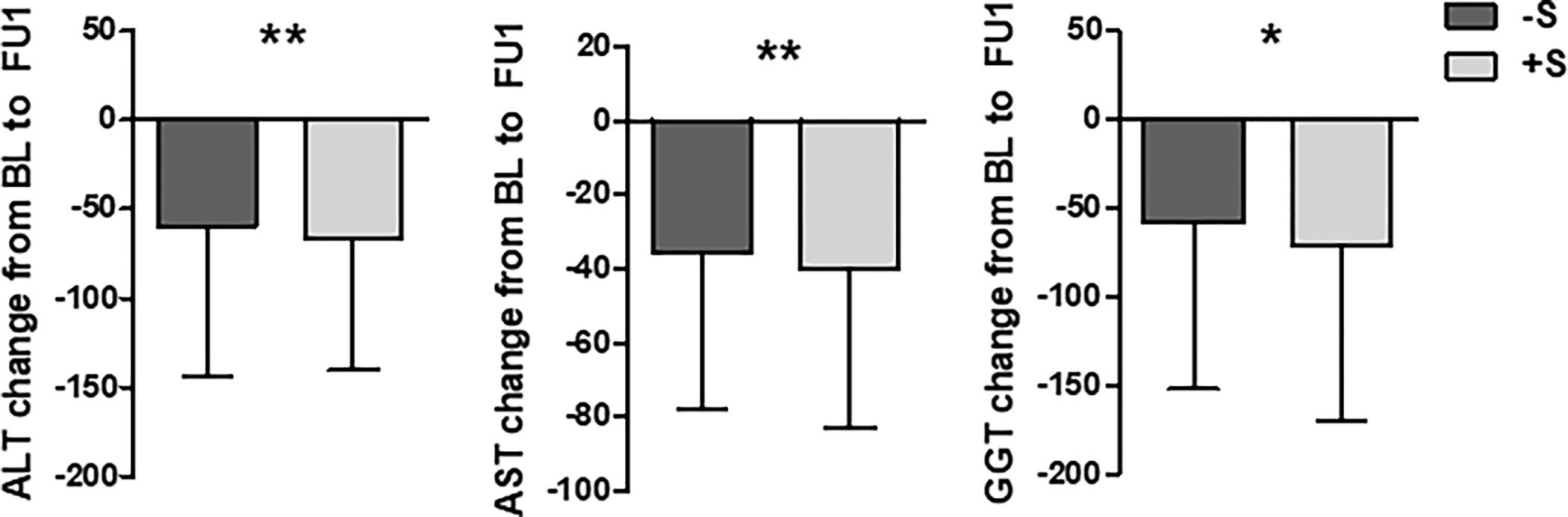
Change of serum liver enzymes from BL to FU1 for HCV patients with steatosis (+S) and without steatosis (-S) and without fibrosis progression. Mean ± SD. * = p < 0.05, ** = p < 0.01.

A significant decrease of ALT, AST and GGT from BL to end of antiviral treatment (EOT) was observed for all patient groups (-S +/- fibrosis progression as well as +S +/- fibrosis progression in all GT as well as GT 3 and non-GT 3 patients) reflecting the effect of antiviral treatment. When analyzing the changes in liver enzymes from BL to FU1 with pairwise comparisons of groups patients +S—fibrosis progression had a larger decrease of ALT, AST and GGT compared to patients -S—fibrosis progression (**Fig 2**). The more pronounced the decrease of ALT, AST and GGT in +S patients -fibrosis progression could reflect an improvement of concomitant NAFLD in these patients. In order to complete the results of this analysis a significant larger decrease of ALT was also observed for +S—fibrosis progression compared to -S + fibrosis progression (mean decrease ± SD: -66.0±74.2U/L vs. -51.5±68.0U/L; p<0.001) and for AST a significant difference in decrease was observed for -S + fibrosis progression vs. -S—fibrosis progression (mean decrease ± SD: -39.5±57.1U/L vs. -35.6±42.1U/L; p<0.05).

A higher reduction of liver enzymes after antiviral treatment was observed in patients with concomitant liver steatosis and without fibrosis progression.

### Analysis of baseline BMI together with liver enzymes might reflect underdiagnosis of NAFLD

**Fig 3:**
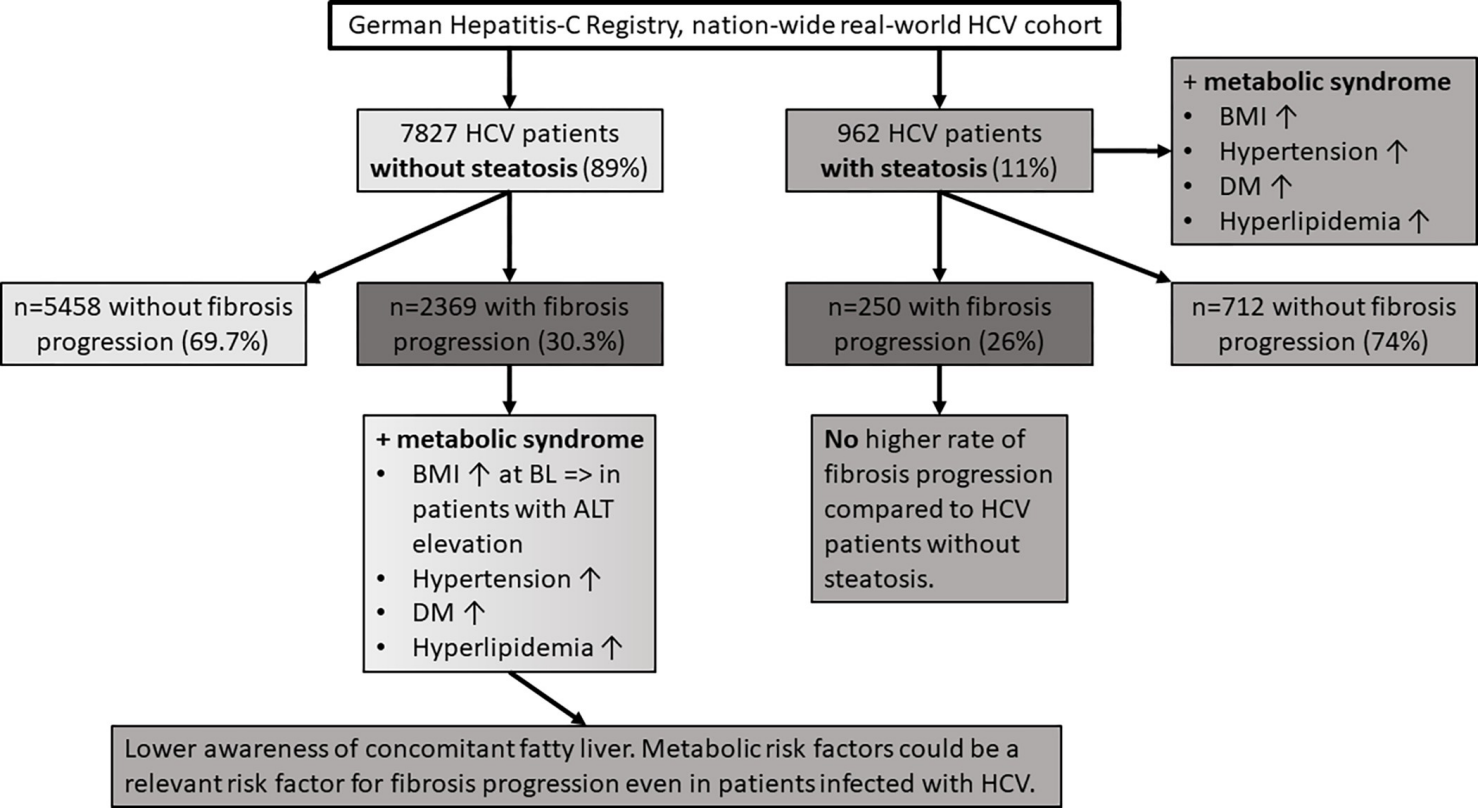
Overview of fibrosis progression and metabolic comorbidities in patients +S and -S.

At BL visit HCV patients -S + fibrosis progression had a significantly higher BMI compared to HCV patients -S—fibrosis progression (BMI mean ± SD; -S + fibrosis progression: 26.0 ± 4.9kg/m^2^; -S—fibrosis progression: 25.4 ± 4.3kg/m^2^, p<0.001). This significant difference in BMI at BL was observed in patients with elevated ALT at BL (BMI mean ± SD; -S + fibrosis progression/ALT elevation: 26.3 ± 4.8kg/m^2^; -S—fibrosis progression/ALT elevation: 25.7 ± 4.2kg/m^2^, p<0.001), but not in patients with normal ALT values at BL (BMI mean ± SD; -S + fibrosis progression/normal ALT: 25.0 ± 4.7kg/m^2^; -S—fibrosis progression/normal ALT: 24.6 ± 4.4kg/m^2^, p = 0.39). These findings might indicate a lower awareness and missed diagnosis of NAFLD in this HCV cohort, as HCV patients -S + fibrosis progression had a higher BMI at BL compared to patients -S—fibrosis progression. Furthermore, HCV patients -S + fibrosis progression had a significantly higher frequency of hypertension, DM and hyperlipidemia in comparison to HCV patients -S—fibrosis progression (**Table 3**) strengthening the hypothesis of underdiagnosed NAFLD. An overview of these findings is depicted in **Fig 3**. HCV patients without diagnosed steatosis and with fibrosis progression had more metabolic risk factors compared to patients without fibrosis progression.

**Table 3:** Metabolic features in HCV patients -S with and without fibrosis progression.

HCV patients -S % (n)			
* *	with fibrosis progression	without fibrosis progression	p-values
* *	(n = 2369)	(n = 5458)	
**BMI (MW±SD)**	26.0 ± 4.9	25.4 ± 4.3	< 0.001
**Hypertension % (n)**	27.8 (658)	20.4 (1111)	< 0.001
**DM % (n)**	14.7 (348)	7.1 (386)	< 0.001
**Hyperlipidemia % (n)**	2.6 (62)	1.9 (101)	< 0.05
**ALT (U/L) (MW±SD)**	85.5±71.1	85.9±78.0	0.827
**AST (U/L) (MW±SD)**	78.9±56.9	61.4±44.3	< 0.001

### No difference in SVR in HCV patients with and without steatosis

HCV patients +S and -S had no significant difference in SVR to antiviral treatment (96.3%, identical for both groups). However, HCV patients +S and -S + fibrosis progression had a significant lower SVR compared to HCV patients +S and -S—fibrosis progression (-S—fibrosis progression 97.5% vs. -S + fibrosis progression 93.5% (p<0.001), +S—fibrosis progression 97.5% vs. +S + fibrosis progression 92.8% (p<0.01)).

### No difference in HCC prevalence for HCV patients with steatosis

The HCC frequency showed no difference in HCV patients +S and -S over time. In patients -S HCC occurred in 0.4% (n = 30) at end of antiviral treatment, 0.4% (n = 29) at FU24w, 1.2% (n = 41) at FU1y, 1.3% (n = 32) at FU2y. HCV patients +S had a HCC frequency of 0.1% (n = 1) at end of antiviral treatment, 0.5% (4) at FU24w, 1.1% (n = 1) at FU1y, 1.2% (n = 3) at FU2. One can speculate that the patient numbers in the HCV patient group +S are not sufficient to analyse the contribution of NAFLD in HCC development in HCV patients.

## Discussion

NAFLD and chronic HCV infection are the leading liver diseases worldwide and hepatic steatosis is often observed as a comorbidity in patients with chronic HCV infection. In this study the clinical course of NAFLD in HCV patients undergoing DAA therapy was analysed in a large, prospectively enrolled nation-wide registry cohort (the German Hepatitis-C Registry). Fibrosis progression was analysed in HCV patients with steatosis (+S) and without steatosis (-S) during almost exclusively successful viral clearance and clinically diagnosed steatosis does not seem to contribute to significant fibrosis progression in this large cohort. No significant difference was observed in clinical endpoints such as SVR and HCC incidence. The surprisingly low prevalence of steatosis in this cohort seems to reflect a low awareness towards NAFLD particularly as an accompanying disease. This may have led to underdiagnosis of NAFLD in HCV patients, which should have been more frequently detectable as HCV patients -S + fibrosis progression presented more metabolic co-components such as a higher BMI, higher frequency of hypertension, DM and hyperlipidemia compared to patients -S—fibrosis progression.

NAFLD is closely related to the metabolic syndrome with a high prevalence of obesity, type 2 DM and dyslipidemia [18]. In HCV GT 1 infection hepatic steatosis is linked to features of the metabolic syndrome such as BMI, visceral obesity, insulin resistance, type 2 DM, low HDL-cholesterol levels [2]. In this analysed cohort HCV patients +S (all GT as well as GT 1 only) presented more often components of the metabolic syndrome such as higher BMI, higher frequency of DM, hypertension and dyslipidemia compared to patients -S strongly indicating a concomitant metabolic liver disease.

Regarding fibrosis progression HCV patients +S did not show a higher rate of fibrosis progression in this analysis, but even a lower rate of fibrosis progression compared to patients -S. This effect was especially observed for GT 3 patients, but not for non-GT 3 patients (**Fig 1**). HCV GT 3 has the highest prevalence of steatosis when compared to other HCV genotypes and is a unique entity in chronic HCV infection [1,6,19]. HCV GT 3 modulates host lipid metabolism and seems to influence unique mechanisms of fat metabolism and transportation within hepatocytes [20]. Studies support the association of higher grades of steatosis with higher rates of fibrosis progression, but the burden of data does not support pathogenic evidence for enhanced direct viral mediated fibrogenesis in GT 3 infection [19]. In this real-world cohort hepatic steatosis was more frequent in GT 3 patients compared to non-GT 3 patients, but still with a much lower rate of only 16.7% (199/1190) compared to the literature of up to 86% (5). These obvious differences can be explained by the diagnostic modality for steatosis assessment since most references from the literature rely on histological steatosis grading whereas steatosis assessment in times of elastometry fibrosis staging is mostly based on ultrasound as in our study. Furthermore, one can speculate that this significant difference between fibrosis progression in HCV GT 3 patients +S and -S could be explained by a different attitude on NAFLD as a concomitant disease to viral hepatitis, particularly since the latter is eventually characterized by increased echogenicity in ultrasound as well.

Liver enzymes were significantly decreased form BL to EOT for all patient groups (+S +/- fibrosis progression and -S +/- fibrosis progression). A rapid decline in liver enzymes after HCV treatment with DAA stands mainly for an improvement in necroinflammation [21,22]. In this cohort HCV patients +S–fibrosis progression showed a larger decrease of ALT, AST, GGT from BL to FU1 compared to patients -S–fibrosis progression. One obvious explanation for this difference in a longer time period after antiviral treatment could be changes in lifestyle under regular supervision of DAA treatment leading to the amelioration of concomitant metabolic steatosis. Like in the placebo arms of randomized clinical trials, these findings highlight the sole impact of regular clinical visits even without specific metabolic intervention.

In a small patient subcohort with available TE data a significant reduction of TE value from BL to FU1 was observed for HCV patients -S with and without fibrosis progression as well as for patients +S and without fibrosis progression. Reduction of liver stiffness after DAA treatment in chronic HCV infection is observed at the end of the treatment period as well as in the FU period up to 24 and 48 weeks after EOT [19,22–24]. Early changes of liver stiffness are rather due to reduced necroinflammation and to a lesser extent to reduction of fibrosis especially in advanced liver disease [25]. Reduction in hepatic steatosis after antiviral treatment is often observed using mainly CAP measurements or evaluation by magnetic resonance imaging-determined proton density fat fraction (PDFF) [26–28]. Liver stiffness over time was reduced non-significantly in our analysis of +S patients with fibrosis progression, but a limited number of only 12 subjects available for TE analysis from BL to FU1 largely limit the interpretation of this data. In this specific patient group concomitant metabolic liver disease may prevent liver stiffness reduction to some extent. Considering the real-world setting of this study no non-invasive data for changes in liver steatosis over time was available in sufficient number.

The prevalence of clinically diagnosed hepatic steatosis in this real-world cohort was lower as expected. This can be explained by an ultrasound-based, non-invasive diagnosis of steatosis, which leads on one hand to a high number of recruited patients, but on the other hand a lower sensitivity for steatosis detection. In a detailed analysis of HCV patients -S with and without fibrosis progression -S patients with fibrosis progression presented significantly more often components of the metabolic syndrome (DM, hypertension and dyslipidemia) compared to -S patients without fibrosis progression. Furthermore, HCV patients -S with fibrosis progression had a higher BMI in comparison to -S without fibrosis progression. This significant difference in BMI was mainly observed for patients -S with ALT elevation, but not for patients with ALT in normal range. Taken together these findings lead to the assumption that in patients -S with fibrosis progression a concomitant NAFLD was underdiagnosed. In a large primary-care record study of 17.7 million adults of four European countries the same diagnostic gap for NAFLD was observed with a much lower rate of at best ~1/6 and more likely only ~1/12 of the estimates based on cohort data [29]. Randomly selected patient data (n = 10 826 456) from Medicare in the US demonstrate likewise that NAFLD is grossly underdiagnosed in real-world setting and in a US cohort of 7033 subjects more than 95% of subjects with suspected NAFLD were unaware of having a liver disease [30,31]. As the lack of awareness and diagnosis of NAFLD *per se* is common, one can assume that the diagnosis of coexistence of NAFLD in chronic HCV patients is probably even lower and explain the low records of hepatic steatosis in this real-world cohort study. Formally, the presence of hepatic steatosis or even steatohepatitis is needed to define NAFLD in HCV patients and the reference standard to diagnose these patterns is still by histology [15]. Here, steatosis is mainly diagnosed by ultrasound with the known limitations for hepatic fat content of less than 30% [32]. Thus, the diagnosis of hepatic steatosis in our large real-world data is only a weak indicator for the real presence of concomitant NAFLD, especially lower grades of steatosis, and is a limitation of our study due to the real-world setting. The final interpretation of HCC development as a clinical endpoint is likewise limited in this analysis due to the low patient number in the group of HCV patients +S. This may explain different findings from recent other cohorts [33,34]. Nevertheless the overall incidence of HCC in non-cirrhotic NAFLD patients is low and no specific recommendations can be based on epidemiological data from large cohorts [35].

Due to the nature of this large real-world cohort there are some limitations in our study. At first not all patients -S had an ultrasound without steatosis at the date of inclusion. There are patients without any available ultrasound and no known diagnosis of steatosis or diagnosed steatosis by ultrasound or histology who are treated as patients -S for this analysis. In the absence of histology as the gold standard of steatosis detection no analysis of true negative HCV -S patients is possible in this study. Fibrosis progression over time was defined by combined non-invasive parameters such as ultrasound, TE and lab scores. No repeated histology was available for a more exact assessment of fibrosis progression. As all patients had antiviral treatment, the natural course NAFLD as a concomitant disease to persistent HCV infection could not be analysed, since HCV has been eradicated in more than 98% of patients. However, preexisting structural damage by HCV may still affect the clinical course of mostly untreated NAFLD. Therefore, our analysis focused on the awareness and impact of clinically diagnosed hepatic steatosis with a background of former HCV infection in a real-world setting. These limitations are due to the real-world setting of a large register study primarily focused on HCV and could not be avoided in this NAFLD oriented analysis.

In this present study clinically diagnosed steatosis does not seem to contribute to significant fibrosis progression in a large real-world cohort of HCV patients. This finding may be relevant and different from other “natural cause” studies, since these patients are prone to pre-existing liver damage by HCV for variable time and extent. The association of fibrosis progression and BMI in those patients without diagnosed NAFLD, however, points towards a role of metabolic cofactors in disease progression in this particular setting. The surprisingly low prevalence of steatosis in this cohort could reflect a lower awareness and diagnosis of NAFLD in HCV patients. As NAFLD is the most common cause of chronic liver disease, it is important to raise awareness of metabolic liver disease not only as unique but also as concomitant liver disease amongst physicians to early identify especially patients at risk. Regular follow-up and attempts to treat NAFLD even after successful HCV clearance may be more important than currently perceived in clinical practice.

## Abbreviations

BL: baseline
DAA: direct antiviral agents
DM: diabetes mellitus
FU: follow-up
GT: genotype
HCC: hepatocellular carcinoma
HCV: hepatitis C virus
NAFLD: Non-alcoholic fatty liver disease
SVR: sustained virological response rate
TE: transient elastography
+S: with steatosis
-S: without steatosis
